# Managing Long-term Orofacial Contractures and Microstomia Through Intraoral Stretching

**DOI:** 10.1093/jbcr/irae123

**Published:** 2024-07-27

**Authors:** Lori Ann Arguello, Kathleen Mary Kerr

**Affiliations:** Center for Speech Pathology, Department of Otolaryngology, University of Texas Medical Branch, Galveston, TX, USA; Center for Speech Pathology, Department of Otolaryngology, University of Texas Medical Branch, Galveston, TX, USA

**Keywords:** facial burn, microstomia, orofacial contractures, oral splinting

## Abstract

Microstomia and orofacial contractures continue to garner interest regarding the effectiveness of treatment methodologies utilized to impact functional change. Oral splints are an accepted tool in the management of microstomia. However, the concepts of which oral splints to use, timing of initiation, and duration of treatment have not gained a consensus. This article reviews approaches to oral splinting and an alternative intraoral approach using splints designed to provide a graded, low load, multidirectional, and prolonged stretch specifically in facial burn survivors including those with mature scars. Two participants participated in a trial using oral splints placed inside the mouth at established contracture points. Participants were requested to use the splints for 1 h twice daily. Participants were photographed weekly producing 9 facial expressions, and distance between 13 facial landmarks was measured to evaluate change in tissue length. Numerical changes observed from beginning to end of the trial indicate positive and negative alterations, signifying lengthening or shortening of tissue. Negative changes denote reduction in distance between endpoints, while positive changes signify an increase. Participants verbalized functional improvements in oral motor and psychosocial function posttreatment. To date, oral splints can be custom fabricated for each individual patient. However, few oral splints are created to provide multidirectional stretch focusing on problem areas across the mid and lower face. The intraoral splints and regimen described here have the capability of providing a stretching intervention that could be applicable in various stages of burn recovery.

## INTRODUCTION

Orofacial contractures and microstomia, common challenges in burn injury survivors, have traditionally been managed using horizontal and circumferential oral splints, in combination with surgical and other nonsurgical interventions. The application of splints in burn care evolved from the principle of maintaining anticontracture positions to preserve tissue length and prevent loss of range of motion during the healing process.^1^ Multidisciplinary burn care teams prioritize early intervention, often implementing positioning and splinting protocols for affected body parts immediately upon admission to maximize functional recovery. However, the timing and methodology of oral splinting for orofacial contractures and microstomia remains debated, with consensus lacking regarding the appropriate types of splints, initiation timing, and duration of treatment.^2,3^ Oral splinting is frequently delayed until scar maturation or until orofacial contractures are readily apparent.^4^

Numerous oral splint designs have been proposed in the literature, each tailored to specific treatment objectives. In 2003, Dougherty and Warden conducted a comprehensive review of oral appliances created between 1972 and 2002. They identified 37 different devices that provided either a horizontal, vertical, or circumoral stretch.^5^ In one example, the oral splint described by Yotsuyanagi and Sawada^6^ introduced a moldable plastic splint to prevent microstomia during intubation and postextubation utilizing a stretch in the horizontal plane. Al-Qattan et al.^7^ described a customized silicone block for stabilizing and stretching oral commissures postsurgery for commissure release. Wust^8^ introduced the modified dynamic mouth splint, incorporating active and passive components for improving mouth mobility. This study by Wust is one of few that engaged a participant who initiated the treatment 22 months postburn with positive results documented in a short treatment period, 21 days. Rumbach et al.^9,10^ and Clayton et al.^11^ incorporate oral splinting as part of a standard treatment plan for orofacial contractures and microstomia. Despite these advancements, and extensive reviews such as the one conducted by Dougherty and Warden^5^ regarding their benefits and limitations, consensus on optimal oral splinting strategies and the timing of their use remains elusive.

Circumferential oral splints, like those discussed by Clayton et al.,^11^ offer benefits in stretching oral tissues away from contracted positions to prevent or treat microstomia. However, the circumferential oral splint may pose challenges for placement in severe cases, potentially necessitating surgical intervention before nonsurgical treatment can commence. Concerns also exist regarding the potential exacerbation of contractures in specific facial areas such as the philtrum, upper and lower vermillion borders, and mentolabial junction when the range in these areas is reduced while the circumferential splint is in place. The reported outcomes in the study by Yotsuyanagi and Sawada^6^ stated maintenance of both horizontal range of motion (HROM) and vertical range of motion (VROM) through the early application of their moldable splint applied while the patient was still intubated. Upon further review of posttreatment photographs, the splint did not appear to address areas that remained contracted such as the philtrum and mentolabial junction. With these concerns in mind, the investigators advocate for oral splints which are adaptable to the directionality of the required stretch and capable of contouring to facial tension lines to promote effective tissue lengthening and functional improvement.

This concept of directionality of the contracture and stretch is particularly pertinent for the facial burn survivor. In contrast to microstomia cases, these patients can also experience retraction of the skin and muscles around the mouth, which exposes the teeth and negatively impacts lip closure for functions such as intelligible speech, straw drinking, and retention of food while chewing.^5,11,12^ The same need for stretching of contracted skin and muscles applies, but the oral splint must be adaptable for directionality of the stretch and one which can contour to facial skin tension lines.^13^ Stretching along the direction of tension lines (Langer’s lines) helps to orient collagen fibers in the same direction within the dermis. It is also important to note that the muscles of facial expression originate from or insert into the skin.^13^ This is important to consider when looking at dynamic movement of the face as overlying contracted skin could be a limiting factor for muscle movement.

In addressing the complexity of facial movement, it becomes evident that a multidirectional approach to oral splinting is necessary.^5^ A 2009 review by a consensus summit, Richard et al.^14^ called for inclusion of an intraoral approach to microstomia intervention in the acute care stage, and also suggested that an emphasis on prolonged stress of the affected tissue may be required, compared to the brevity of active range of motion and facial exercises. In an effort to create this multidirectional and prolonged stretch, oral splints with the potential to target multiple points of contracture and accommodate varying stages of recovery were developed by the investigators.^15^ To this end, this study discusses the use of these novel three-dimensional oral splints designed to address specific contracture points in facial burn survivors using an intraoral methodology. Here data is presented for two facial burn survivors who are no longer considered to be in the acute or early rehabilitation phases of their recovery as the burn injuries were remote (ie, occurred 17 years ago for one participant and 30 years ago for the other). The investigators seek to determine if the use of the oral splints presented here results in changes in tissue length (lengthening vs shortening of skin and muscles) for targeted locations around the mouth for a chronic or long-standing impairment in facial range of motion caused by a facial burn injury.

## METHODS

### Study setting and approval

This study was conducted in a university hospital clinic setting with approval from the institutional review board. The institutional review board determined the use of the intraoral splints to be minimal risk. A comprehensive description of the oral splints and their intraoral placements can be found in the article by Arguello et al.^15^ AI assistance in the form of ChatGPT^16^ was utilized as a tool during writing of this manuscript for wording of article content only and not for data collection, analysis, or production of images.

### Intervention description

**Figure 1.**
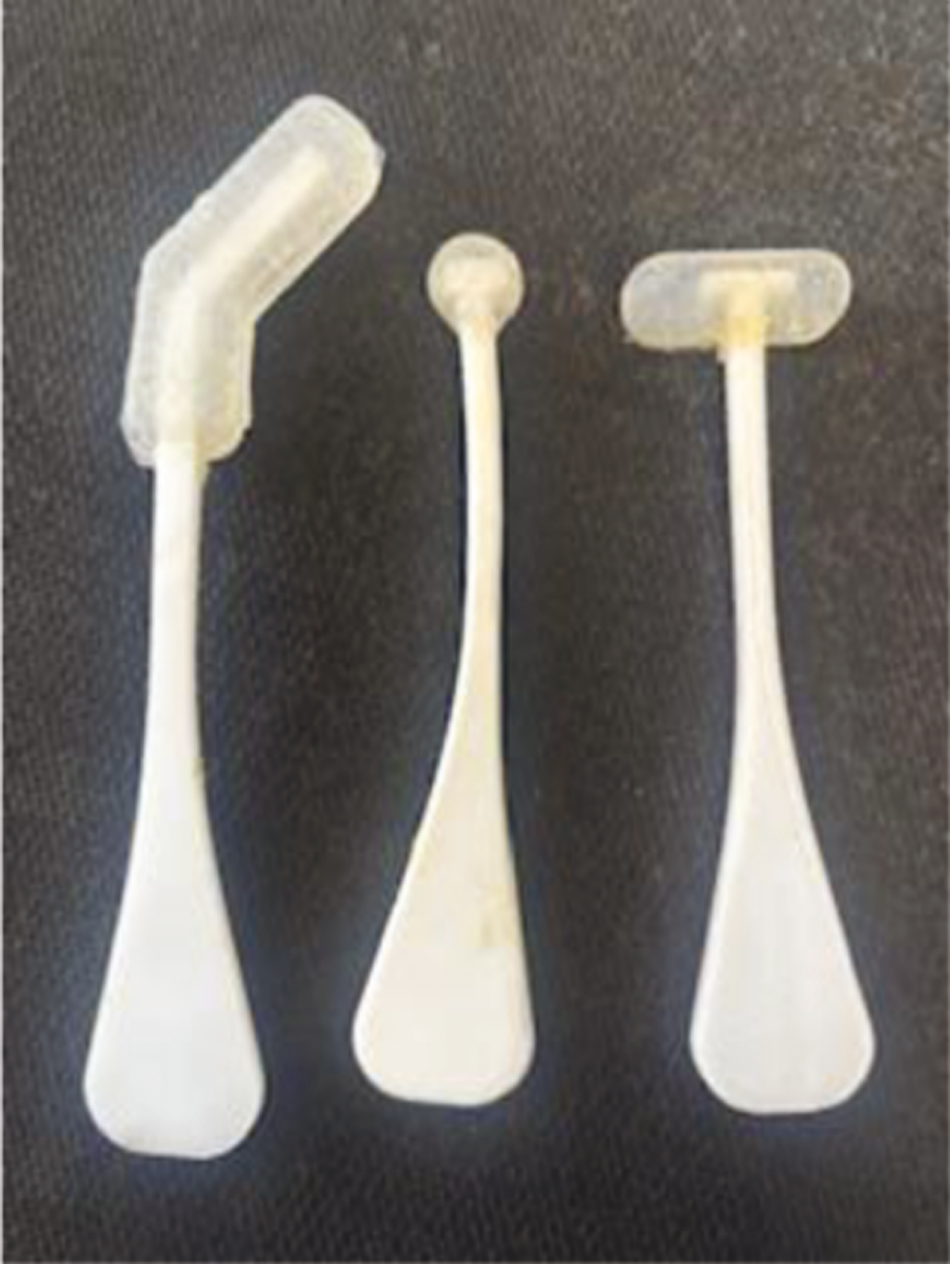
Photograph Showing the Intraoral Splints

**Figure 2.**
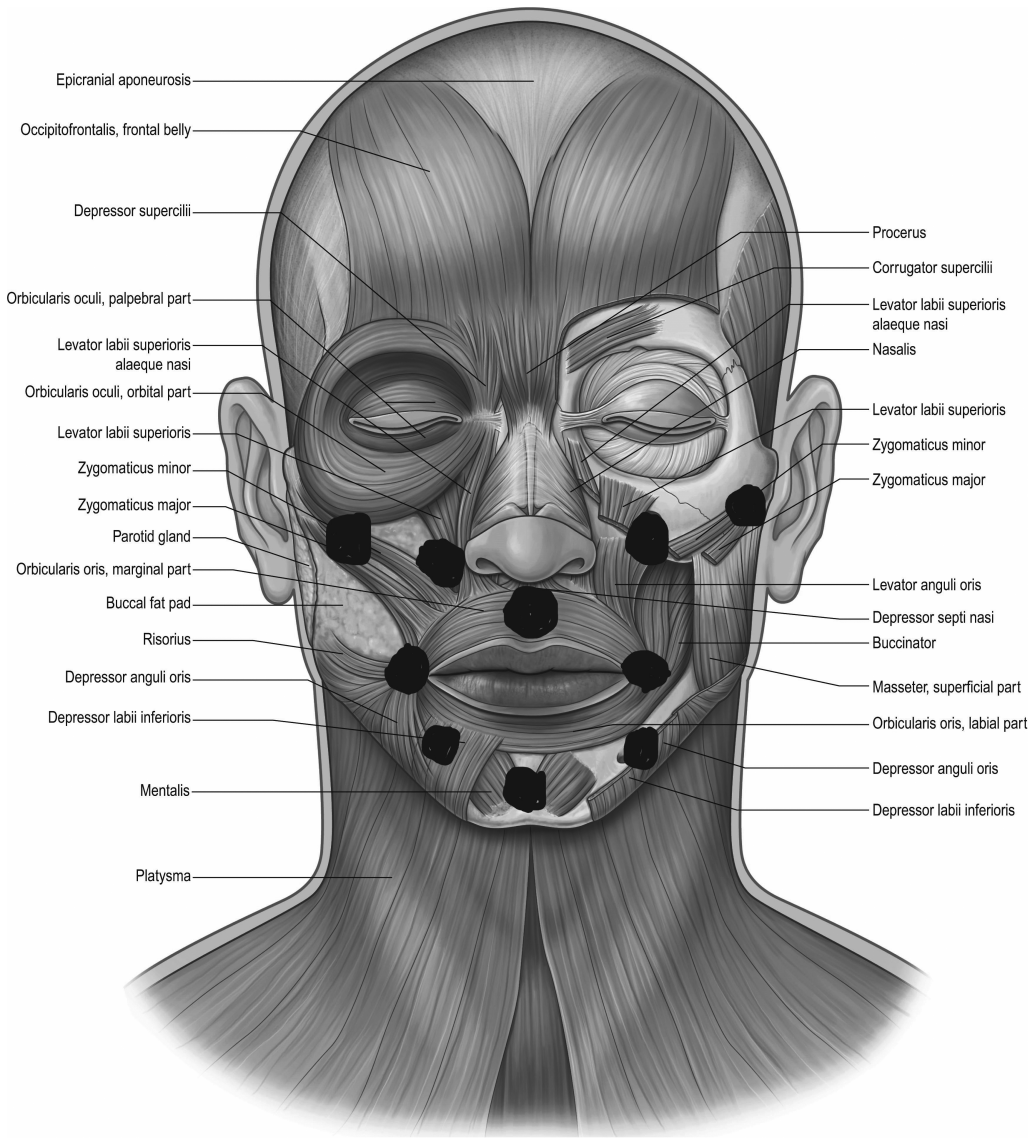
Common Contracture Points After a Facial Burn

**Figure 3.**
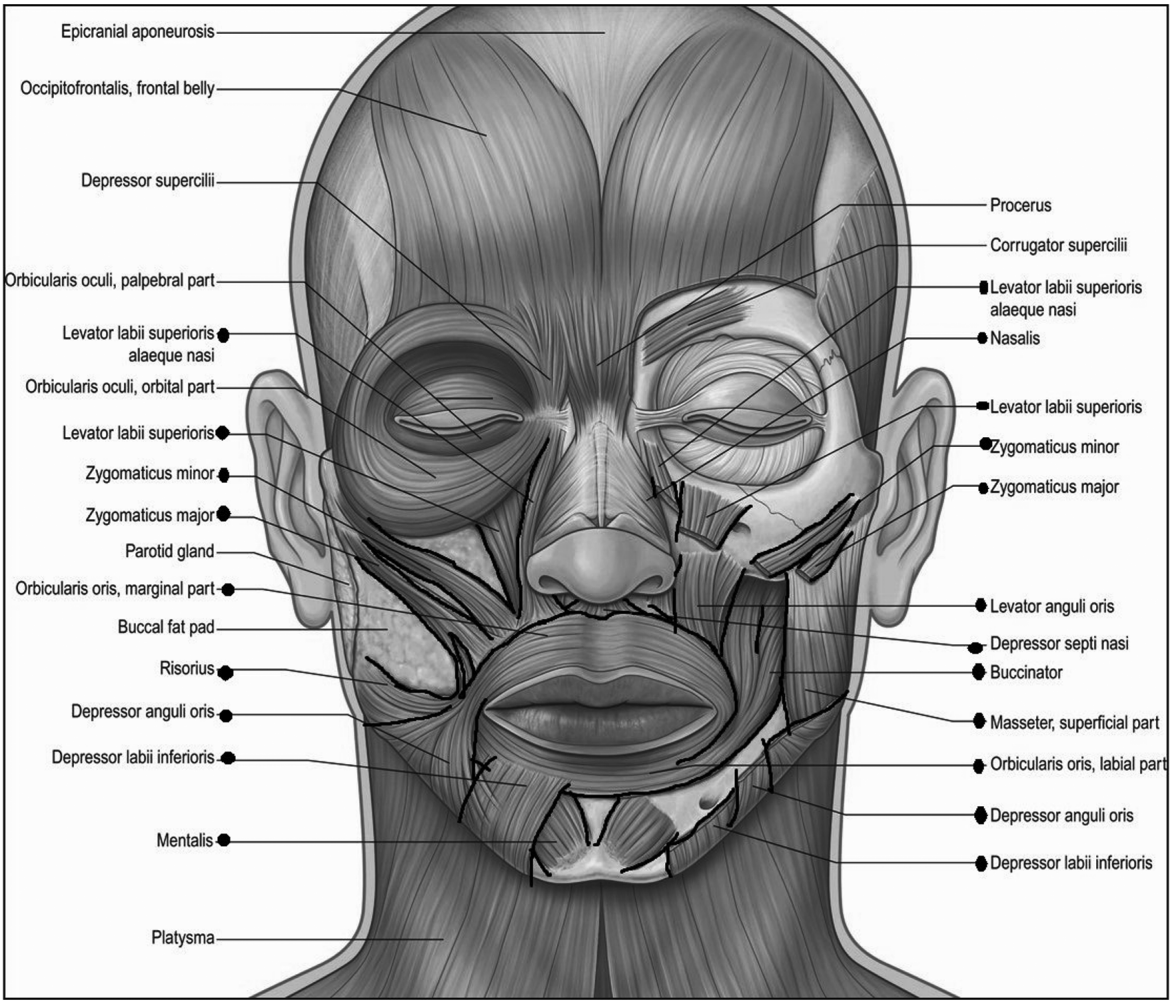
Muscles Potentially Impacted by Placement of the Intraoral Splints

**Figure 4.**
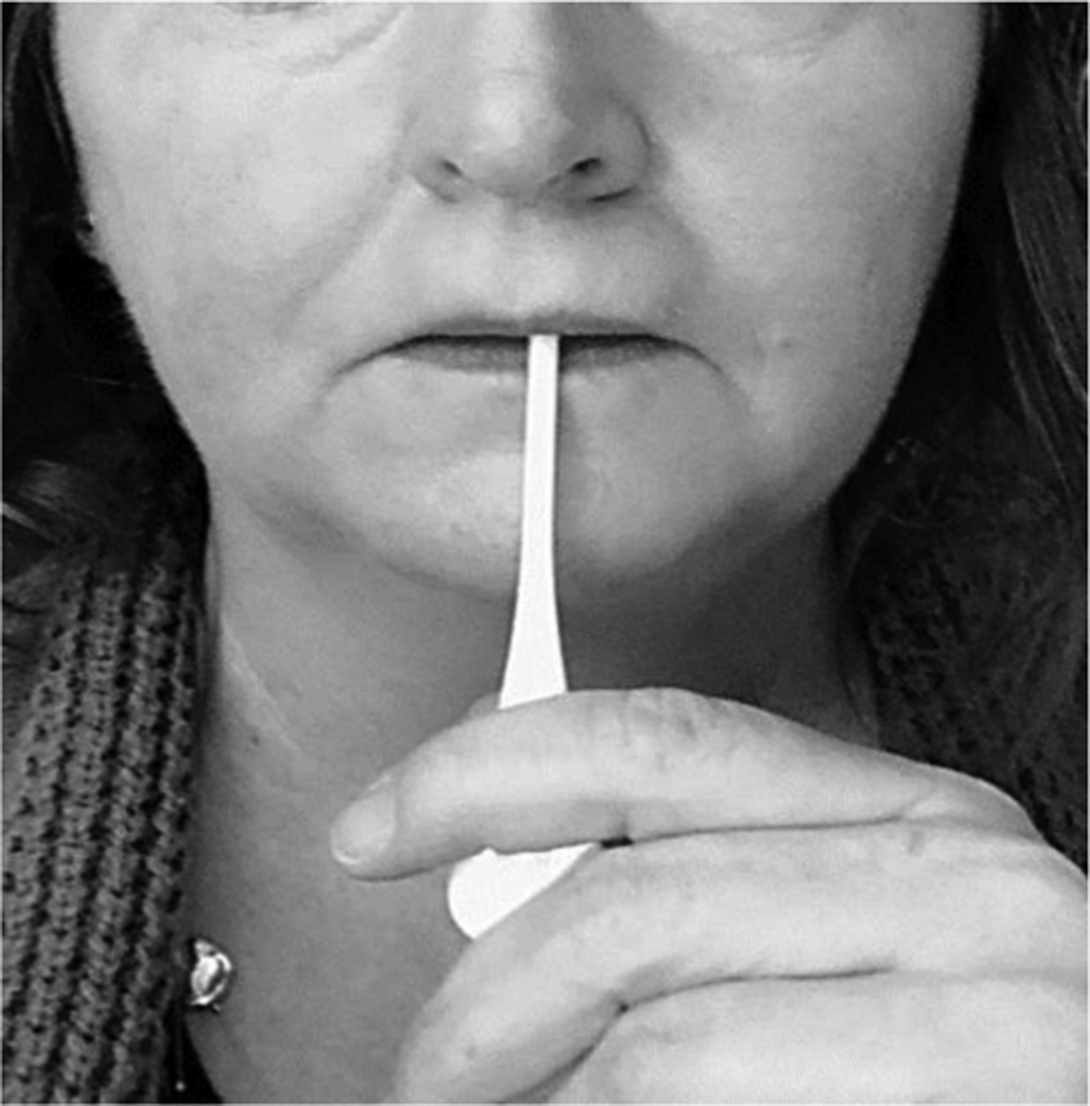
Example Intraoral Placement of the Bar Shape Splint Underneath Upper Lip

The intraoral splints were intentionally designed to administer a low-load, prolonged stretch to common contracture points across the mid and lower face. These contracture points were identified based on anatomical considerations and previous research findings.^15,17–20^ To ensure effectiveness, the splints were specifically engineered for intraoral placement, allowing for targeted stretching of facial skin and muscles. A detailed description of splint construction can be found in the 2023 article by Arguello et al.^15^ The oral splints are shown in Figure 1. Common contracture points and possible muscles and overlying skin impacted by these intraoral splints are superimposed on the image “Facial Musculature”^21^ (Figures 2 and 3, respectively). Notably, the splints were designed with an intraoral placement to deliver a multidirectional stretch beyond the conventional vertical and horizontal planes, thereby addressing the complex dynamics of facial movement. The importance of a low load, prolonged stretch has been described in the Physical Medicine and Rehabilitation literature as being necessary to achieve lasting lengthening of a muscle.^22,23^ The investigators observed a need for a splint with capability of reaching and providing an outward stretch to multiple areas across the mid and lower face. An example placement with one of the intraoral splints is shown in Figure 4. This shows the bar-shaped splint placed underneath the upper lip, with the handle outside the mouth.

### Study duration and participant evaluation

The study was originally planned for 8 weeks; however, due to unforeseen circumstances, final measurements for Participant 1 were taken at 5 weeks. Participant 2’s data was assessed at weeks 5 and 8 to evaluate any ongoing changes throughout the full 8-week program.

Participants were scheduled for weekly in-person or virtual visits during which photographs were taken, participant feedback was recorded, and adjustments to splint shape, size, and placement were made based on investigator-identified progress and participant feedback. The participants were photographed weekly, facial landmarks were identified, and distance between chosen endpoints was measured in the photographs to track changes posttreatment. Measured distances included horizontal distance between outside of the nares, pupil to outside of the nares (right and left), pupil to outside corner of the lip (right and left), superior edge of the philtrum to outside corner of the lip (right and left), nares to outside corner of the mouth (left and right), length of the philtrum, superior border of the upper lip to inferior border of the lower lip, horizontal distance between outside corners of the lips, and inferior border of the lower lip to inferior tip of the chin.^17–20^ VROM was measured as superior border of the upper lip to inferior border of the lower lip, rather than the traditional method of distance between central incisors or distance between inner lips, in order to better capture the presence of and change in lip inversion or eversion. The above-mentioned data points were collected in photographs of nine different facial expressions which were: at rest, gentle smile, broad smile with the lips closed, broad smile showing teeth, say “eee,” say “ooo,” pucker as if drinking through a straw, open mouth wide, and wrinkle your nose.^17–20^ A customized 2D facial measurement software was created in-house and used to collect measurements between the chosen facial landmarks during this study, as referenced in the 2023 study by Arguello et al.^15^ The participants were requested to use the selected intraoral splint for 1 h twice per day. Each week the participants provided daily logs recording the length of time the selected intraoral splints were utilized. Table 1 includes information regarding the selected splint shape, size, placement location, and amount of time used. Finally, the Facial Disability Index (FDI) was used to capture participants’ perceptions of disability.^24^ The FDI was completed by each participant at study start and study end. Further detail can be found in the article by Arguello et al.^15^

**Table 1.** Use of the Intraoral Splints for Participants 1 and 2

Week	Splint shape	Splint size	Location	Average time with splint in place	Sessions per day
Participant 1					
1	L shape	Large	Bilateral lower cheek along mandible	30–60 min	2
2	Bar shape	Medium	Lower lip	60 min	2
3	Ball shape	Large	Left commissure	30–40 min	2
4	Ball shape	Large	Left and right commissures	40 min	2
5	L shape	Medium	Right upper cheek along maxilla	40 min	2

Participant 2					
1	L shape	Medium	Left upper and lower cheeks along maxilla and mandible, right lower cheek along mandible	30 min	1
2	Bar shape	Medium	Upper and lower lip	30 min	2
3	L shape	Large	Bilateral lower cheeks along mandible	30 min each location	2
4	L shape	Medium	Left upper and lower cheeks along maxilla and mandible, right upper andlower cheek along mandible	60 min	2
5	L shape and bar shape	Large	Bilateral lower cheeks along mandible with placement closer to commissures	30–60 min	2
6	L shape	Large	Left lower cheek along mandible	30–60 min	2
7	L shape	Large	Left lower cheek along mandible with placement closer to commissure	30–60 min	2
8	N/A	N/A	N/A	N/A	N/A

### Participants

Each participant signed an informed consent form and indicated they did not plan to undergo any other treatments during the course of this study, including the use of pressure garments, additional oral splints, surgical interventions, massage, or laser treatments.

#### Participant 1

Participant 1 was a 42-year-old female who experienced a flame burn injury to her face and neck as a child. Little is known about her early treatment course as the burn injury occurred more than 30 years ago and the participant had limited recall of her treatment history. Her primary complaint was for lack of space between lips and gums due to contractures causing tightness, facial asymmetry, pressure on her teeth from contracted tissue, and the inability to have orthodontic care. Observations of oral motor function, facial animation, and facial symmetry included functional but decreased mouth opening, contractures of the upper lip, philtrum, and mentolabial junction causing tightness of this tissue across her upper and lower teeth, and asymmetrical scarring and pulling of the facial skin with changes in facial expression. Baseline oral opening was measured at 30 mm (noting the difference in measurement of oral opening as above). Selected splints and intraoral placements for Participant 1 are shown in Table 1.

#### Participant 2

Participant 2 was a 43-year-old male with a history of flame burn injury which occurred 17 years ago from wildfires. He reported to have a 75% total body surface area burn with significant midface and lower facial burns. He reported lower lip eversion in an “at rest” posture and unintentional mouth opening with neck extension. These difficulties persisted despite a history of neck exercises and use of pressure garments and a remote history of skin grafting to the neck. He complained of drooling, difficulty brushing his teeth, and difficulty with facial movements. Observations of oral motor function at the initial visit included a baseline mouth opening of 50 mm, measured as a distance from superior edge of the upper vermillion border to inferior edge of the lower vermillion border (measured at midline). During physical examination during the first visit, the participant was noted to have increased tightness on the lower right side of the face. He had scar tissue bands on the left side of the nose extending to the left commissure and horizontally to the cheek area, as well as on the right commissure that extended inferiorly to the jawline. The participant verbalized that his bottom lip felt “thicker,” and this equated to reduced pliability and motion. Selected splints and intraoral placements for Participant 2 are shown in Table 1.

## RESULTS

The numerical changes observed from the beginning to the end of the study, as depicted in the provided tables, indicate both positive and negative alterations, signifying lengthening or shortening of tissue between identified facial landmarks or endpoints. Negative changes denote a reduction in the distance between endpoints, while positive changes signify an increase. Given the diverse recovery trajectories and the individual nature of scar tissue, cross-participant comparisons were not conducted. While data was collected for 13 facial landmarks across 9 facial expressions, the investigators report data for the key expressions: “at rest,” “open mouth wide,” and “broad smile showing teeth,” along with a discussion of specific facial landmarks crucial for assessing symmetry and function of the orofacial musculature.

**Figure 5.**
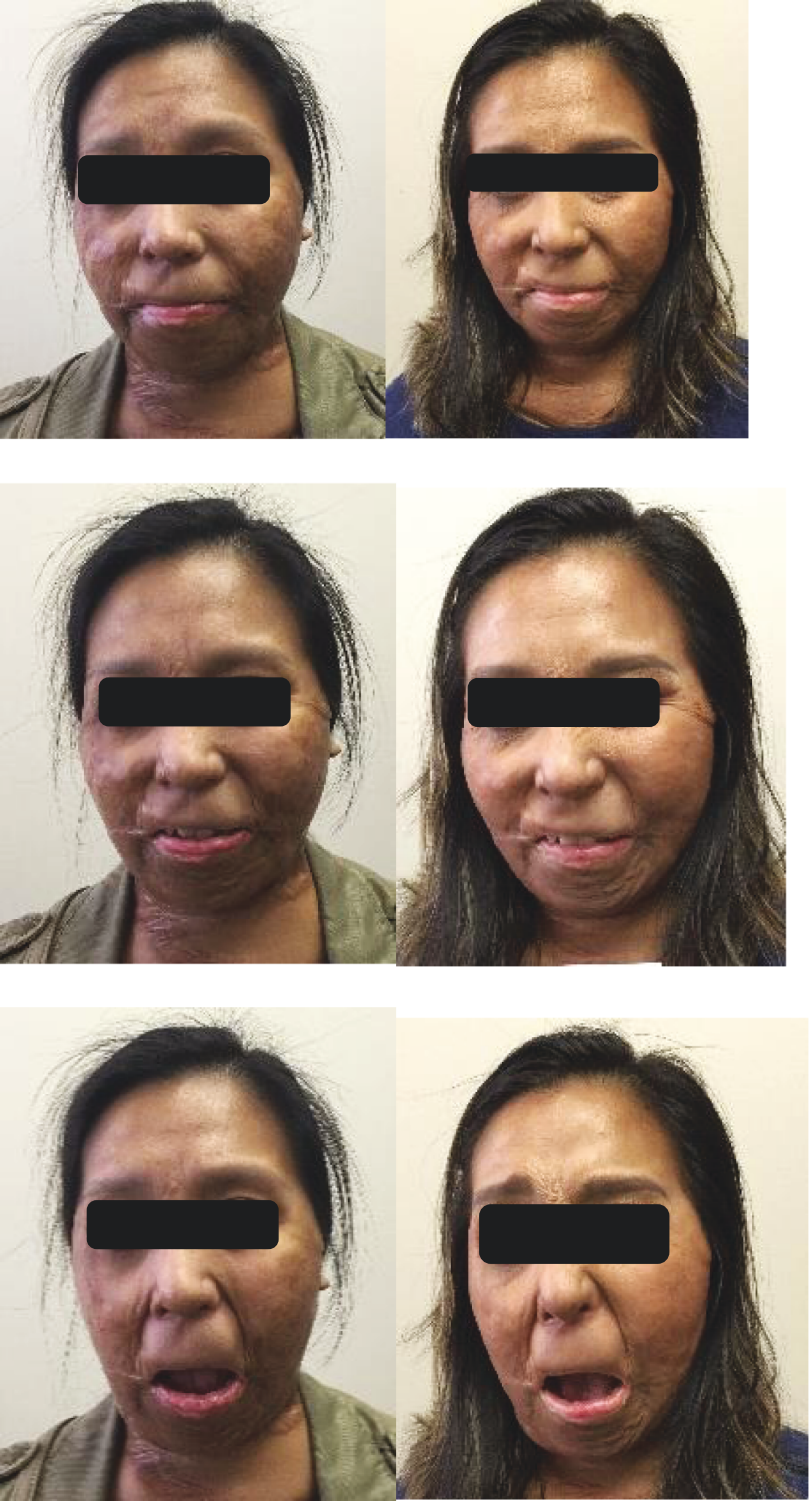
Photographs of Participant 1 from Week 1 (Left) and Week 5 (Right) of Study Treatment. Facial Expressions Were At Rest (Above), Broad Smile Showing Teeth (Middle), Open Mouth Wide (Bottom)

**Figure 6.**
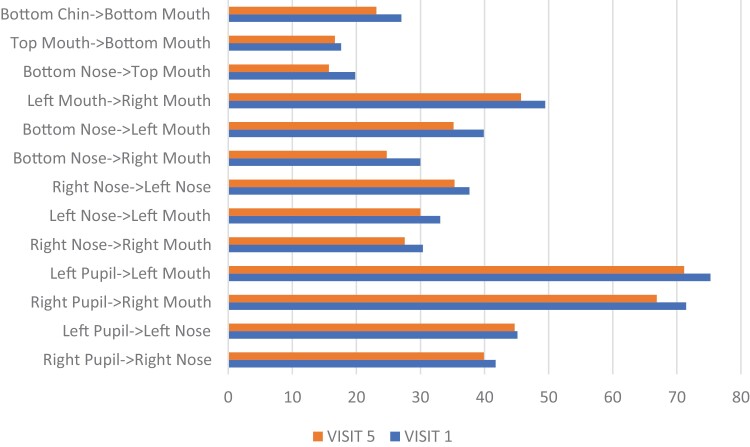
Participant 1 Posttreatment Change with the Expression “At Rest”

**Figure 7.**
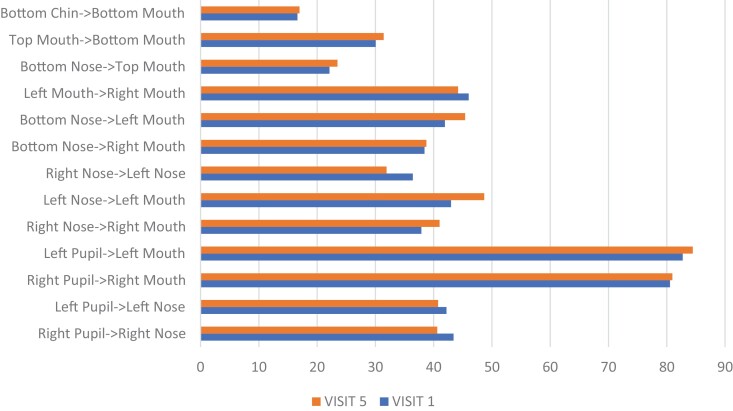
Participant 1 Posttreatment Change with the Expression “Open Wide”

Participant 1 demonstrated change as shown in Figures 5–7. After 5 weeks of intervention, her mouth opening increased from 30.0 mm to 31.4 mm, as measured from the superior point of the upper vermillion border to the inferior point of the lower vermillion border when performing the expression “open wide.” HROM changed from 46.0 mm to 44.1 mm when performing the expression “broad smile show your teeth,” along with a change in the length of the philtrum from 15.7 mm to 13.9 mm. This demonstrates the importance of understanding and addressing how multiple muscle groups impact targeted facial movements. Change in the measured landmark inferior point of lower vermillion border to inferior point of chin was 27.0 mm to 23.1 mm in the expression “at rest.” Tissue at the lower vermillion border and mentolabial junction is evaluated to assess the length of the overlying skin and interactions of multiple muscles including the mentalis muscle, the depressor labii inferioris muscle, and the orbicular oris, each of which contribute to lip movement, mouth closure, and symmetry in this evaluated “at rest” position.

**Figure 8.**
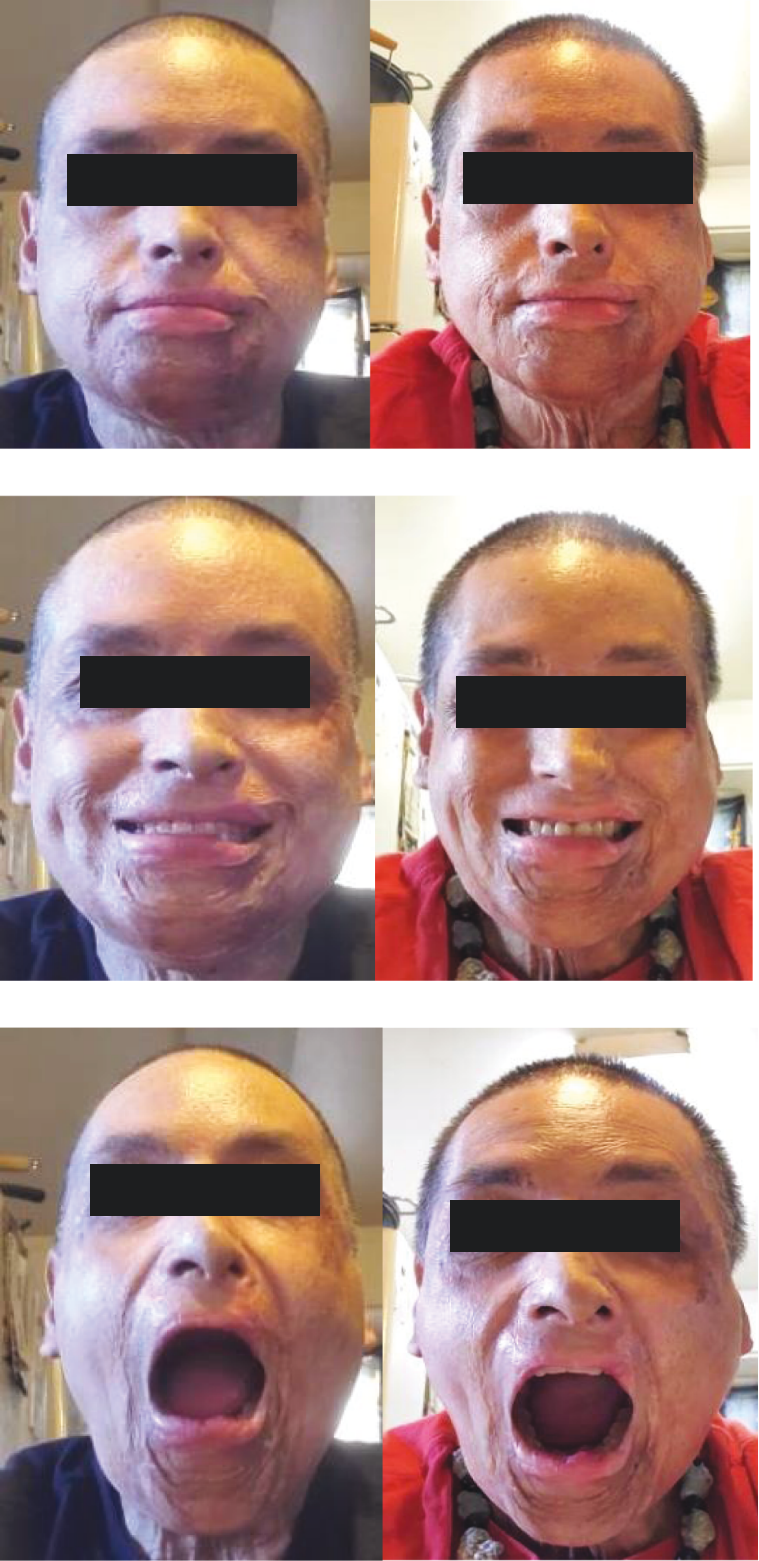
Photographs from Participant 2 from Study Start (Left) and Week 8 (Right) of Study Treatment. Facial Expressions Were At Rest (Above), Broad Smile Showing Teeth (Middle), Open Mouth Wide (Bottom)

**Figure 9.**
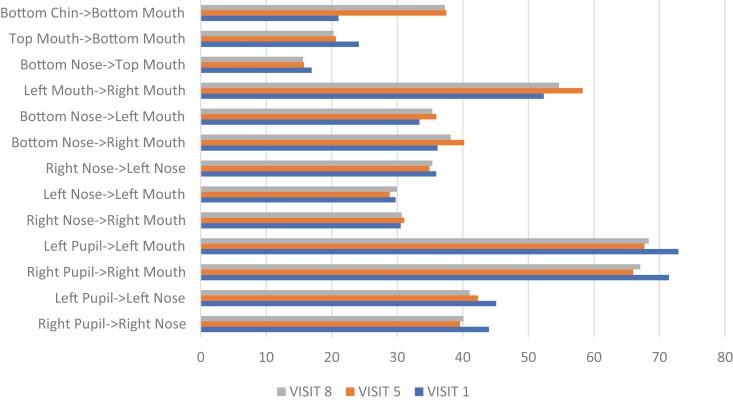
Participant 2 Posttreatment Change with the Expression “At Rest”

**Figure 10.**
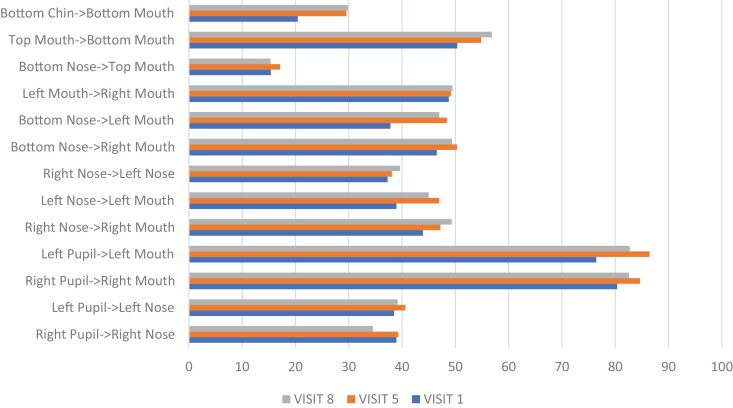
Participant 2 Posttreatment Change with the Expression “Open Wide”

Figures 8–10 show the visual changes from initial to final treatment for Participant 2. The data was evaluated for this participant at both week 5 and week 8 as he was able to complete the full 8 weeks. Participant 2 experienced a positive directional change in mouth opening from 50 mm to 54 mm at 5 weeks and to 56 mm at 8 weeks when performing the expression “open wide.” Again, mouth opening was measured from the superior point of the upper vermillion border to the inferior point of the lower vermillion border at midline. HROM also showed a positive directional change from 58.4 mm to 61.7 mm (week 5) and 62.5 mm (week 8) when performing the expression “broad smile show your teeth.” Change in the length of the philtrum was 16.9 mm to 15.7 mm (week 5) and 15.6 mm (week 8) in the expression “at rest.” A shortening of this tissue in the context of this facial expression reflects a return to a more neutral lip position. Change in the measured landmark of inferior point of lower vermillion border to inferior point of chin was 21.0 mm to 37.4 mm (week 5) and 37.2 mm (week 8) in the expression “at rest,” indicating a lengthening of tissues.

Subjective changes following splinting intervention were captured through verbal report and use of the FDI. Participant 1 reported increased “freedom of movement” and enhanced range in her cheek mobility as seen in the ability to “puff up her cheeks with air.” The physical score from her FDI improved from 22 to 23 at week 5. The social/wellbeing score improved from 28 to 38 at week 5. Total FDI score improved from 177 to 190 (out of 200). Participant 1’s pre- and posttreatment reporting on the FDI demonstrated improvements in facial range of motion and function from much difficulty to little difficulty brushing teeth/rinsing mouth; from waking up a few nights to no longer waking up at night; from facial function keeping her from going out or participating in family/social activities a little bit of the time to none of the time.

Participant 2 verbalized his facial muscles felt “looser,” and he reported increased oral access and ease of brushing his teeth by week 5. This participant also verbalized that the scar bands across his face were more pliable. The physical score from his FDI remained the same at 60 for both week 1 and week 8. His social/wellbeing Score improved from 28 to 40 at week 8. His total FDI score improved from 88 to 100 (out of 200) after 8 weeks of treatment. Participant 2’s reporting on the FDI demonstrated improvements in drinking from a cup and in speech clarity, as well as reduced feelings of isolation, improved teary/dry eyes, and improved sleep.

## DISCUSSION

The cases presented here highlight the ongoing functional challenges caused by the chronic impairments resulting from old facial burn injuries and emphasize the need for innovative treatment approaches to address these long-term challenges. The presented findings contribute important preliminary data towards incorporating use of an intraoral methodology with a focus on multidirectional, low load, and prolonged stretching into treatment protocols for the management of orofacial contractures and microstomia, including cases in which the injury is remote. This study contributes to the necessary discussions of whether chronic impairments caused by a remote facial burn injury can be positively impacted with nonsurgical treatment. The observed changes in tissue length underscore the need for further research into the potential for intraoral stretching to induce functional improvements in orofacial range of motion. Length of time since initial injury date should not be considered a barrier to implementation of nonsurgical treatment methodologies in a facial burn patient with chronic orofacial impairments.

The inclusion of an array of facial landmark measurements in this study emphasizes the need for comprehensive assessment protocols for orofacial contractures and microstomia. While facial measurement software offers precision, manual measurements remain feasible alternatives, allowing for a more thorough evaluation of orofacial function and symmetry. In addition to the traditional VROM and HROM measurements, this study proposes additional measurements including lip inversion or eversion, philtrum length, and distance from lower vermillion border to bottom of chin. These measurements can enhance the burn care team’s assessment of microstomia, as well as the less frequently discussed lip retraction.

### Strengths, limitations, and future direction

Strengths of this study include the utilization of new intervention tools with a focus on an intraoral approach that can impact tissue length and potentially manipulate more than one muscle group at a time. The ability to provide a unilateral or a bilateral stretch to affected portions of the face is also a strength. Review of data during this brief study can continue to provide valuable initial insights into the efficacy of these splints and an intraoral methodology. Limitations of this study include the small sample size, the brevity of the study without long-term outcomes, and the need for further investigation into the length of time needed to produce both an immediate and a long-term, sustained change in orofacial function. The observed additional improvements in facial measurements for Participant 2 from week 5 to week 8 suggest the potential for further gains with extended treatment durations, warranting further investigation into optimal duration, and intensity of the treatment protocol.

Challenges in objectively assessing scar progression highlight the need for multidisciplinary collaboration in burn care management and standardized assessment protocols. Moving forward, there is a pressing need for high-quality research focusing on functional outcomes following intervention^25^ with the development of a standardized orofacial assessment protocol^14^ which can be used by the entire burn care team to identify and quantify changes in orofacial range of motion beyond VROM, HROM, and burn scar depth across the long-term treatment span of a facial burn survivor. Data-driven assessments of baseline status and progress are crucial for optimizing treatment strategies and improving outcomes for facial burn survivors.

## CONCLUSION

Ongoing research and discussions among the members of the multidisciplinary burn care team are needed with regard to optimal care of facial burns and their associated functional and aesthetic challenges. Continued pursuit of alternative treatment methodologies outside of surgical intervention is needed to provide affordable and impactful care across a broad spectrum of facial burn patients, including chronic facial burn patients, while considering factors influencing access to care such as geographic distance from a burn center or treating burn care therapist. Readily available oral splints with clear protocols for use can assist in this endeavor.
